# Left ventricular rupture after leaflet grasping in transcatheter edge-to-edge mitral valve repair

**DOI:** 10.1093/ehjcr/ytad614

**Published:** 2023-12-08

**Authors:** Ayano Yoshida, Kazuki Mizutani, Genichi Sakaguchi, Gaku Sr Nakazawa

**Affiliations:** Division of Cardiology, Department of Medicine, Kindai University Faculty of Medicine, 377-2 Ohno-Higashi, Osakasayama, Osaka 589-8511, Japan; Division of Cardiology, Department of Medicine, Kindai University Faculty of Medicine, 377-2 Ohno-Higashi, Osakasayama, Osaka 589-8511, Japan; Department of Cardiovascular Surgery, Kindai University Faculty of Medicine, Osaka, Japan; Division of Cardiology, Department of Medicine, Kindai University Faculty of Medicine, 377-2 Ohno-Higashi, Osakasayama, Osaka 589-8511, Japan

## Case description

**Figure 1:**
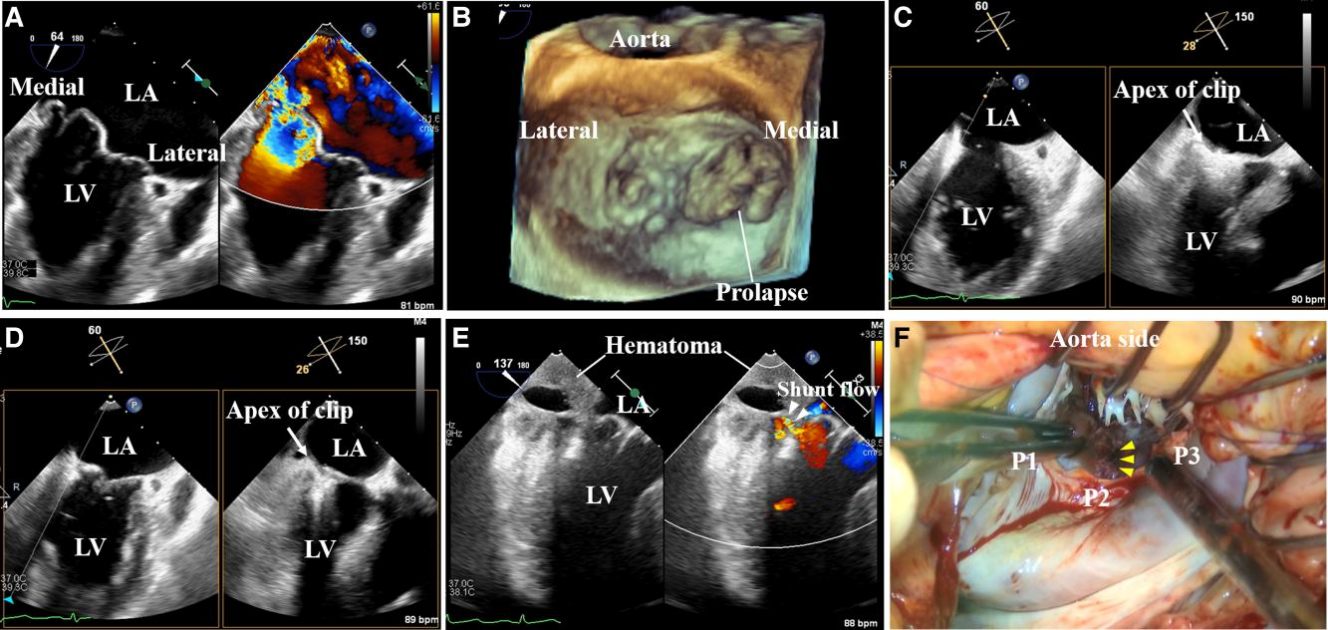
(*A*) Severe eccentric mitral regurgitation from medial of A2–P2 to A3–P3 area. (*B*) Three-dimensional image of the mitral valve showing prolapse of A2, A3, and P3. (*C*) Just after MitraClip gripper down. Arrows indicate the leaflets on the clip. (*D*) Once the clip was completely closed, the clip was sinking into the myocardium (arrows). (*E*) A shunt flow was observed just below the mitral valve annulus on the left ventricular posterior wall, which formed a haematoma around it. (*F*) Myocardial fissure from the mitral annulus of the P3 to the left ventricular apex (arrows). LA, left atrium; LV, left ventricle; P, posterior.

A 92-year-old man presented with worsening shortness of breath (NYHA III). Echocardiography revealed sliced reduced left ventricular (LV) systolic function (ejection fraction: 55%), preserved right ventricular function with moderate tricuspid regurgitation, and severe mitral regurgitation due to prolapse of A2, A3, and P3 (effective regurgitation area, 1.66 cm^2^; regurgitant volume, 176 mL) (*Figure 1A* and *B*). The length of the posterior mitral leaflet (PML) was 12.3 mm, the anterior mitral leaflet (AML) was 32.2 mm, and the prolapse gap was 7.2 mm. Since he had no history of open chest surgery but was elderly, had high surgical risk (STS score for mitral valve repair, 15.9%), our heart team opted for transcatheter edge-to-edger repair (TEER). First, we tried to grasp the medial scallop using the MitraClip G4 XTW system (Abbott Medical, CA, USA). However, since the grasping length of PML was inadequate, we performed individual P3 grasping after A3 grasping and gained sufficient grasping of both leaflets. Immediately thereafter, the systolic blood pressure suddenly dropped, and circumferential pericardial effusion appeared. Although it is an after-the-fact verification, the clip was sinking into the myocardium as the clip closed (*Figure 1C* and *D*).

A shunt flow was observed just below the mitral valve annulus on the LV posterior wall, which formed a haematoma around it (*Figure 1E*). Although immediate drainage was performed, we decided to convert to surgical repair due to persistent active bleeding. It was determined that retrieval of the clip would risk exacerbation of the bleeding, and a surgical procedure was performed with the clip detached and in place. An ∼2 cm myocardial fissure was observed from the mitral annulus of the P3 to the LV apex (*Figure 1F* and Supplementary material online, *Video S1*). His blood pressure remained around 50 mmHg under catecholamine administration until he was placed on extracorporeal circulation, but he did not go into cardiac arrest. The myocardial fissure was sutured and covered with a pericardium patch, and mitral valve replacement was performed (Epic 29 mm; Abbott Medical) (see Supplementary material online, *Video S2*). The postoperative course was good, and the patient was discharged alive on postoperative Day 28. There have been no reports of LV rupture by TEER. In this case, it is thought that the PML was shorter than the AML as the tip of the clip on the posterior apical side reached the edge of the valve annulus, which caused it to sink into the myocardium during the closing process of the clip. This is the first report regarding LV rupture during TEER. When TEER is performed on medial or lateral marginal lesions, more care should be taken, such as delineating the extent to which the clip enters the base of the valve leaflet on TEE.

## Supplementary Material

ytad614_Supplementary_Data

## Data Availability

All data and software code on which the conclusions of the paper rely available to readers.

